# Operation of air‐conditioning and sanitary equipment for SARS‐CoV‐2 infectious disease control

**DOI:** 10.1002/2475-8876.12238

**Published:** 2021-07-02

**Authors:** Takashi Kurabuchi, U. Yanagi, Masayuki Ogata, Masayuki Otsuka, Naoki Kagi, Yoshihide Yamamoto, Motoya Hayashi, Shinichi Tanabe

**Affiliations:** ^1^ Department of Architecture Faculty of Engineering Tokyo University of Science Tokyo Japan; ^2^ School of Architecture Kogakuin University Tokyo Japan; ^3^ Department of Architecture Faculty of Urban Environmental Sciences Tokyo Metropolitan University Tokyo Japan; ^4^ College of Architecture and Environmental Design Kanto Gakuin University Yokohama Japan; ^5^ School of Environment and Society Tokyo Institute of Technology Tokyo Japan; ^6^ Faculty of Engineering Tokyo Polytechnic University Kanagawa Japan; ^7^ Faculty of Engineering Hokkaido University Sapporo Japan; ^8^ Department of Architecture Waseda University Tokyo Japan

**Keywords:** airborne transmission, building equipment, COVID‐19, SARS‐CoV‐2, ventilation

## Abstract

It is still undetermined if the main infection route of severe acute respiratory syndrome coronavirus 2 (SARS‐CoV‐2), the virus that leads to coronavirus disease 2019 (COVID‐19), is infection through droplet, contact, or airborne transmission. However, confined spaces with poor ventilation are cited as a risk factor for group outbreaks, and there is growing interest in the effects of intervention through the appropriate operation of air‐conditioning and sanitary equipment to reduce the risk of airborne transmission. This study first offers an outline of the characteristics of the novel coronavirus disease and the cluster outbreak case reports that have been clarified until now. Subsequently, we describe the appropriate operating conditions for building equipment that are effective in reducing the risk of infection and also highlight specificities for each building use based on the guidance provided by healthcare institutions and with reference to the standard recommendations by Western academic societies related to building equipment.

## Graphical abstract

Effective use for building equipment for SARS‐CoV‐2 infectious disease control

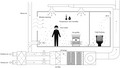

## 1 Introduction

The condition of a closed space with poor ventilation, which corresponds to one of the “Three Cs: crowded places with many people nearby; close‐contact settings such as close‐range conversations; closed spaces with poor ventilation” that can result in a high risk of outbreaks, as indicated by the Japanese Ministry of Health, Labour and Welfare in the Novel Coronavirus Expert Meeting conducted on March 9, 2020,^1^ was the first condition to be associated globally with the possibility of airborne transmission leading to coronavirus disease 2019 (COVID‐19). In response to this, the Society of Heating, Air‐Conditioning and Sanitary Engineers of Japan (SHASE) and the Architectural Institute of Japan (AIJ) issued an emergency presidential discourse titled “Role of ventilation in the control of COVID‐19 infection” on March 23, 2020,^2^ where they described the characteristics of this virus infection and an overview of ventilation methods. Furthermore, they conducted a question and answer session on ventilation titled “Role of ventilation in the control of COVID‐19 infection” on March 30, 2020,^3^ where they provided a general overview of ventilation for the public.

Meanwhile, the Ministry of Health, Labour and Welfare published a document titled “Ventilation to improve closed spaces with poor ventilation in commercial and other facilities”^4^ and compiled a leaflet titled “For managers of commercial and other facilities: ventilation methods to improve closed space with poor ventilation”^5^ on March 30, 2020. Among the described ventilation methods, it cannot be guaranteed that a recommended ventilation can reliably prevent infection; however, a closed space with mechanical ventilation does not fall under the classification of a closed space with poor ventilation if it satisfies the condition of an indoor CO_2_ concentration below the threshold of 1000 ppm (i.e., 30 m^3^/h of outdoor air supply per person is secured) as specified by the Act on Maintenance of Sanitation in Buildings, or if the air changes per hour are greater by two times or more when windows are open (i.e., windows are fully opened for several minutes at least once every 30 min). It is recommended that the required ventilation rate is maintained even in restaurants that do not fall under the specified building types described in the Act on Maintenance of Sanitation in Buildings; moreover, the necessary ventilation rate can be achieved by reducing the number of people in the room if the ventilation rate is insufficient.

On March 29, 2020, the World Health Organization (WHO) initially stated that the primary infection routes of this virus were droplet transmission and contact transmission,^6^ with airborne transmission occurring only under special medical procedures. Countermeasures in such scenarios included social distancing of 1‐2 m or more, wearing masks, regularly washing hands, and disinfecting contact items and indoor surfaces. Subsequently, there were many cases of outbreaks occurring in closed spaces such as a bus in Zhejiang Province,^7^ a restaurant in Guangzhou,^8^, ^9^ and a call center in Seoul,^10^ which could not be explained without assuming airborne transmission. Therefore, the WHO changed its policy on July 9,^11^ stating that although the primary infection routes of the coronavirus disease had not changed, the possibility of infection in a congested space with insufficient indoor ventilation due to the discharge of aerosol particles from exhaled breath and the inhalation of a certain amount of these aerosol particles in combination with droplet transmission could not be ruled out. Consequently, the WHO recommended “Avoid the Three Cs,” which was originally translated from Japanese as countermeasures for a COVID‐19 infection.^12^

Based on the findings obtained to date, aerosol particles that contain the virus and are ejected by the infected person may remain active for more than 3 h in a suspended condition in air, and the active virus is not easily observed in patient isolation rooms with a high air change rate. Therefore, it is almost certain that maintaining the highest possible ventilation rate will lead to a decreased infection risk, under the assumption that the ventilation recommended by the Ministry of Health, Labour and Welfare is properly implemented.

Heating, ventilation, and air‐conditioning (HVAC) systems are normally operated with the objectives of optimizing resident comfort, workplace productivity, and energy conservation; however, it is speculated that these objectives could be downplayed for a certain amount of time and the operation could be adjusted to reduce infection risk as a countermeasure against COVID‐19.

The Federation of European Heating, Ventilation and Air Conditioning Associations (REHVA)^13^ and the American Society of Heating, Refrigerating, and Air‐Conditioning Engineers (ASHRAE) have also presented similar recommendation policies^14^; however, their positions differ in terms of handling the return air. REHVA acknowledges that there have been no cases of infection caused by the transport of aerosols directly through the ducts of ventilation systems; however, they have recommended that the return air (air that returns from the air‐conditioning space to the air conditioner and is recirculated) be stopped based on the “as low as reasonably achievable” principle. Meanwhile, ASHRAE has taken the position that using an effective air filter for an HVAC system that uses return air will lead to a reduction in the infection risk.

This paper is based on “Operating of air‐conditioning equipment and other facilities for severe acute respiratory syndrome coronavirus 2 infectious disease control (revised second edition)” as compiled by the authors and was published on the SHASE homepage on September 7, 2020,^15^ along with the addition of subsequent findings.

### 1.1 Content of this review

1 Recent findings on the infection route and infectivity of SARS‐CoV‐2
2 Risk of infection due to the return of air
3 Droplet size, air filter capture performance, and maintenance management
4 Natural ventilation by opening windows
5 Visualization of indoor air environment
6 Benefits of face masks
7 Flushing the toilet with the lid closed and the enforcement of ventilation
8 Adjustment of temperature and humidity by air conditioning
9 Effective use of an air purifier
10 Germicidal ultraviolet (GUV)
11 Notes of caution for resuming equipment
12 Hand hygiene and peripheral hand‐washing equipment
13 Recommended operating methods for building equipment for each building application
  a Principles of measures to reduce risk of airborne diseases
  b Offices (central air‐conditioning system)
  c Offices (unit air‐conditioning system)
  d Residences
  e Schools
  f Cinemas /theaters
  g Izakaya /Karaoke bars

## 2 Recent Findings on the Infection Routes and Infectivity of SARS‐CoV‐2

Between January and February 2020, a study was conducted on virus activity in the isolation rooms of three patients at a COVID‐19 outbreak center in Singapore^16^. The air change rate of the isolation rooms was 12 air changes per hour (ACH), and the rooms were sterilized daily. Surface and air samples were collected from the isolation room of one patient before cleaning and from the other two patients after cleaning. Reverse transcriptase‐polymerase chain reaction was used to examine the presence of SARS‐CoV‐2 in the samples. The former patient had symptoms of upper respiratory lesions but no fever or diarrhea, and the latter two patients had moderate symptoms of coughing and fever.

The virus was detected in several surface samples collected from the isolation rooms of the patients before cleaning, including samples from the bathroom and the stools of the patient. The virus was not detected in the air samples, but it was detected on the surface of the air exhaust outlets. The virus was not detected in any of the samples collected from the isolation rooms after cleaning. These study results showed that indoor surfaces in the residences of COVID‐19 patients are extensively contaminated; however, sufficient disinfection and sterilization can remove the contamination. Furthermore, aerosol particles containing the virus can move to exhaust outlets, but sufficient ventilation effectively dilutes the virus concentration in the air. Finally, the stools of infected patients may be an infection route.

A research team at the National Institutes of Health also conducted a comparative experiment on the activity maintenance of the severe acute respiratory syndrome (SARS) coronavirus (SARS‐CoV‐1) and SARS‐CoV‐2 in aerosol particles and on surfaces such as plastic, stainless steel, copper, and cardboard.^17^ The results showed that SARS‐CoV‐2 in the form of aerosol particles that are smaller than 5 μm in diameter in a chamber had a decreased infectious titer but maintained activity for 3 h, which was the duration of the experiment. The activity was also maintained on stainless steel and plastic for 3 days, cardboard for less than 1 day, and copper for less than 4 h. Overall, the results showed that SARS‐CoV‐1 and SARS‐CoV‐2 had similar activity maintenance characteristics over time. This study suggests the possibility of COVID‐19 infection through aerosol particles containing the virus and airborne transmission. Evidence of airborne transmission has also been reported for SARS‐CoV‐1.^18^

Chin et al.^19^ measured the stability of SARS‐CoV‐2 in various environments and obtained the following results. A virus transport medium with a log reduction of the median tissue culture infectious dose per milliliter (log TCID_50_/mL) of SARS‐CoV‐2 of 6.8, which indicates the infectious titer, was maintained constant for 14 days, and its infectious titer was measured at the time points of 1, 5, 10, and 30 min, 1, 3, 6, and 12 h, as well as 1, 2, 4, 7, and 14 days. While considering the factor of temperature, high stability was observed at 4°C, with hardly any change in infectious titer until 14 days later. Infectivity was maintained for 7 days and 24 h at 22 and 37°C, respectively, but the infectious virus was no longer detected after 30 and 5 min at 56 and 70°C, respectively. While considering the factor of surface types, the infectious virus was detected on paper and tissue paper surfaces for up to 30 min, but was not detected after 3 h. The infectious virus was detected in one out of three wooden and cloth surfaces for one day; however, the virus was not detected in any of the samples on the second day. Meanwhile, the infectious virus was detected on banknotes for up to two days (but were not detected after 4 days) and stainless/plastic surfaces for up to 4 days (but were not detected after seven days). While considering surgical masks, although the infectious titer was 1/1000 of the initial value, an infectious virus was present on the interior for up to 4 days (log TCID_50_ was 5.88 at 0 min, 2.47 after 4 days, and was no longer detected after 7 days) and on the exterior for up to seven days (log TCID_50_ was 5.78 at 0 min and 2.79 after 7 days).

Next, we discuss the outbreak cases that could be used as a reference when considering measures for HVAC systems. First, we focus on an infection cluster generated within a group at a choir practice in the United States.^20^ A secondary infection case of 53 people (33 confirmed cases, 20 suspected cases) out of 61 people caused by one infected individual following 2.5 h of practice was confirmed on March 10, 2020. In addition to the proximity between the members and the cooperation in cleaning up the chairs, the act of singing itself emitted a large number of droplets, which may have caused the spread of infection. This event led to the identification of risks in live music clubs and karaoke bars.

The cluster infection that occurred at a call center in Seoul, South Korea, in March is important for considering infection prevention measures for office buildings.^10^ A large cluster of 94 workers out of 216 workers was confirmed to be infected in a call center on the eleventh floor of an office/residential building. Based on the results of investigations into the cause of infection, it was understood that there were insufficient infection countermeasures. Thus, there was a small occupied area of 3 m^2^/person with shared desks having low partitions; the staff were convivial and had lunch together while chatting in the meeting room; fingerprint authentication was used for work process management; moreover, the operation of the total heat exchanger was suspended despite the combination of a unit‐type air conditioner and total heat exchanger in the building.

Next, the cluster infection that occurred at a worship event in Zhejiang Province^7^ involved 67 bus passengers traveling to and from the temple on January 19, with 23 confirmed secondary infection cases from a single infected individual that occurred during the 100 min of the round‐trip route. The air conditioning of the bus was operated in a recirculation mode, and there were four open windows; however, the distribution of the secondary infection cases was not closely related to the distance from the primary infected individual and covered almost the entire length, from the front row to the back row of the bus.

Finally, we discuss the cluster infection that occurred at a restaurant in Guangzhou. A family who returned from Wuhan on January 23 and entered Guangzhou had a meal at a restaurant the next day, with one of them developing symptoms on that night. There were nine cases of secondary infection among the guests, which included the table where the family ate and the two adjacent tables, i.e., a total of three tables. The minimum length of overlap in time when the first infected individual and the secondary infection cases were present was 46 min, and camera records in the restaurant showed that the possibility of contact transmission was low. The report that first addressed this event^8^ estimated that the cause of infection was the heating airflow from the fan coil unit attached to the top of the three tables, which formed a circulating airflow between the tables, with droplets from the infected person getting mixed into that airflow, resulting in a travel distance that was longer than the normal scattering distance of droplets.

In a subsequent study,^9^ researchers reproduced the conditions at that time onsite and conducted measurements of ventilation rate as well as simulations of the indoor airflow. The results showed that all the ventilation fans in the restaurant were stopped when the outbreak occurred, with only the fan in the restroom near the entrance in operation; consequently, the air change rate in the restaurant was 0.56‐0.77 ACH, and the outdoor air supply for the 89 customers with a volume of 431 m^3^ was significantly low at 2.7‐3.7 m^3^/h per person. It was concluded that the cause for the spread of infection was the circulating airflow formed by the fan coil air‐conditioning unit and the low dilution of the virus owing to minimal ventilation. This case is important as its results suggest the risks of operating only the air conditioner in a crowded environment in a poorly ventilated state or an environment that is ventilated intermittently rather than continuously.

**FIGURE 1:**
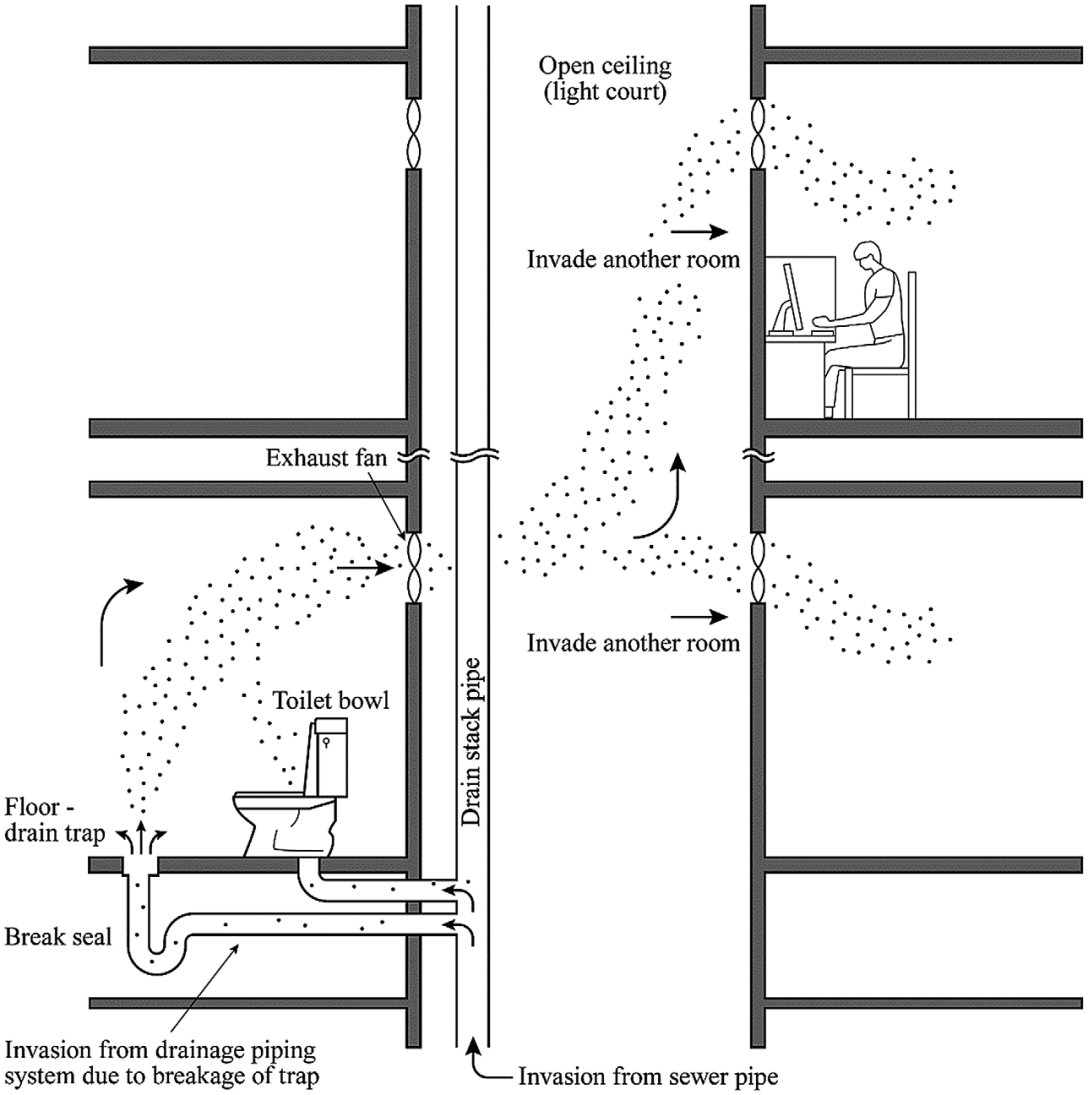
Infection route at Amoy Gardens^24^ (partially modified from original figure)

Next, we considered the infection risk conveyed by virus‐loaded aerosol particles in the toilet plume that occurs in the toilet space and drainage pipes when cleaning the sewage containing infectious viruses or microorganisms, which has long been indicated as an infection route.^21^ As shown in Figure 1, the SARS outbreak at Amoy Gardens in Hong Kong was considered to have occurred because the infectious aerosol particles generated by flushing the stools of an infected person flowed backward through the drainage pipe in the room where the trap seal was broken, and then spread from the indoor environment to the outdoor environment and entered another house by air flow.^22^, ^23^, ^24^, ^25^ It has also been reported that SARS‐CoV‐2 entered and caused infections in a high‐rise apartment building in Guangzhou, China, through the drainage stacks and vents from a trap seal where the sealed water had evaporated in the bathroom.^26^ In Japan, drain trap seals are secured and appropriate vent measures are taken for traps; consequently, the risk of trap seal breakage at the drainage pipe, which could act as a diffusion path for infectious aerosol particles, is considered to be low. However, the active virus has been detected in the stools and urine of COVID‐19 patients^27^, ^28^; therefore, there is a possibility that aerosol particles containing the virus are generated when flushing the stools of infected individuals, which could contaminate the toilet and spread infection by leaking out of the room, although there is currently no evidence of infection through stools or urine.

In particular, a REHVA report^13^ focused on floor drain U‐traps that were connected to drainage pipes and may have been an infection route, indicating that the seal water should be checked and replenished at least once every three weeks. It was also reported both domestically and internationally that viruses were detected in the wastewater of sewage treatment facilities that collect and treat wastewater from buildings during the periods when there were increases in the number of infected people; therefore, there is a need to focus on maintaining the seal water of the trap to prevent the invasion of viruses from the drainage pipe that is directly connected to the sewer.^29^, ^30^

## 3 Risk of Infection Due to Return Air

We also considered the case of the passenger ship Diamond Princess, which was the scene of an outbreak, to analyze the risk of infection in a central air‐conditioning system that circulates return air. Under normal operating conditions, the guest room compartments of the Diamond Princess have a circulating air‐conditioning system with 30% outside air and 70% return air, which is similar to a general central air‐conditioning system.^31^ Therefore, a previous report indicated that the return air of the circulating air conditioner may have contributed to the spread of infection between guest rooms.^32^ To date, three reports have focused on this issue.

Almilaji et al.^33^ compared the symptomatic infection rates in rooms with confirmed infections and those without infections during the isolation period following the start of the quarantine and identified no statistically significant differences, thereby claiming that airborne transmission occurred through the air‐conditioning system. This paper based its arguments on the assumption that air circulation between the guest rooms continued even during the quarantine and isolation; however, on February 5, when the quarantine started, the circulation fan was stopped, fire and smoke prevention dampers were closed, louvers in the doors between the living room and the corridors were closed, and operation of the circulating air‐conditioning system was discontinued; consequently, the assumptions were not in line with the actual circumstances aboard the ship.^34^ Furthermore, the effects of infection routes other than airborne transmission and the exposure prior to the quarantine period were not considered.

Additionally, Azimi et al.^35^ reported the results of numerical simulations of the relative contribution rate of each infection route to the spread of infection on the Diamond Princess. The results showed that the propagation of aerosol particles containing SARS‐CoV‐2 during short‐range contact and over long distances was likely the most dominant infection route for COVID‐19 transmission, even when assuming an air change rate of 9‐12 ACH and no recirculated air inside the ship; thus, it was concluded that measures to prevent the inhalation of small aerosol particles are required in addition to measures against droplet and contact transmission. However, the paper only described the comparison between the actual number of reported cases and the estimated total number of infected individuals and the predicted contribution rate by infection route, and it was not clear whether the infection occurred within the same room or between different rooms; consequently, it was unclear whether the infection was assumed to be caused by air circulation or advection between the rooms. Azimi et al. first listed the lack of available information as a limitation to the modeling approach of their study, resulting in multiple estimates and assumptions being made regarding the transmission characteristics of COVID‐19, individual interactions onboard, ventilation status, and effectiveness of infection control measures during the quarantine period, which resulted in a large degree of uncertainty. Moreover, as a second limitation, constant values and average values were assumed for many of the input values during repeated computations (for example, the rate at which passengers had short‐range contact with other passengers and the amount of droplets/droplet nuclei released were set to be constant for all passengers).

Meanwhile, a report by Xu et al., who also investigated and analyzed the aforementioned case of the Guangzhou restaurant^36^ has been published. Xu et al. analyzed the dynamics and route of infection aboard the Diamond Princess based on the case information and identified that most of the onboard infections occurred in the public areas up to February 5, where normal services were provided prior to the start of the quarantine, with infections occurring only among passengers in the same room when one of them was already infected after February 6; thus, no cross‐room infection was observed between passengers during the quarantine period. Therefore, it was estimated that infection through the central air‐conditioning system (i.e., long‐distance airborne transmission) did not spread the infection, and most of the infections were caused by close contact prior to isolation.

It is speculated that these reports did not adequately reflect the status of circulated air during the quarantine period and did not analyze or consider the impact it would have on the spread of infection aboard the Diamond Princess. As mentioned above, the operation of the circulating air‐conditioning system was suspended during the quarantine period, and the guest rooms were controlled by positive pressure; consequently, there was no recirculation of air between the rooms, and it is unlikely that the air‐conditioning system was the cause of the spread of infection. Clarifying whether the spread of infection was caused by the circulated air requires an investigation of cases where the infection was considered to occur before February 5, which was when the quarantine period began, as well as considering the possibility of droplet and contact transmission. Therefore, the Diamond Princess case does not appear to provide evidence that the return air increases the risk of infection.

Bill Bahnfleth, who chairs the Epidemic Task Force of ASHRAE, said that thousands of papers have been published since the start of the pandemic, and there have been no reports of space‐to‐space infection spreading through air conditioning; conversely, it is considered that the air‐conditioning system provides ventilation and filtration, which reduces the risk of infection.^37^ While acknowledging the possibility of airborne transmission, the WHO has stated that the primary infection route is from person to person^11^; moreover, it has been confirmed that cases that resulted in multiple secondary infections and where ventilation was indicated to be insufficient as an infection preventing measure included the cases where conversations or heavy breathing occurred in a confined space, in addition to spaces where the “three Cs” overlapped.^38^

The infectious virus contained in the return air may spread from one room to another, but its concentrations can be maintained at a sufficiently low level if proper ventilation and air filters are installed. Therefore, the risk of spreading infection between rooms through an air‐conditioning system is considered to be significantly low in a normal air‐conditioned space.

## 4 Droplet Size, Capture Performance of Air Filters, and Maintenance Management

Previous research has reported that the particle size of active droplets derived from the human respiratory system is predominantly < 5‐10 μm.^39^ While considering viruses, Liu et al.^40^ investigated the particle size characteristics of SARS‐CoV‐2 in a hospital in Wuhan city, China, where an outbreak occurred, showing peaks in the submicron (0.25–1.0 µm) and micrometer size (2.5 µm) regions. While considering the infection route, there are certain aspects where engineering and medical perspectives differ when discussing droplet infection or airborne transmission. In engineering, decisions are made by examining the diffusion range of aerosols in the air and the viral activity. SARS‐CoV‐2, which is a fine particle, is suspended in the air for long periods of time and diffuses to distant places by airflow. Furthermore, there is a reasonable possibility that a fraction of the aerosols (which cannot be completely removed) re‐enters the room through the air‐conditioning system. Meanwhile, in the medical field, there is an emphasis on the infectivity of aerosols consisting of droplets and droplet nuclei following their diffusion. That is, the question is whether aerosol particles, including diffused viruses, would immediately lead to an infection. The occurrence of infection is determined by the dose–effect (response) relationship. The dose–effect (response) relationship of SARS‐CoV‐2 is currently unknown. While considering the infectious SARS‐CoV‐2, Santarpia et al. detected SARS‐CoV‐2 generated from patients using a particle‐size measuring instrument at the three stages of <1 µm, 1–4 µm, and >4.1 µm, and reported that five out of six subjects had a peak at <1 µm, and one subject had a peak at >4.1 µm. This study also used TCID_50_ to evaluate the viral activity.^41^ Noti et al.^42^ measured the influenza virus in a simulated hospital room and determined that the composition ratios of the virus at <1 µm, 1–4 µm, and >4 µm were 19.5, 75.5, and 5%, respectively.

Next, the air filter captures suspended particles through the filter media by one or more of the following mechanisms of gravitational settling: inertial impaction, interception, diffusion, and electrostatic attraction. The capture mechanism differs depending on the particle size, with impaction and diffusion having a high‐collection efficiency for large and small particle sizes, respectively; however, the collection efficiency is the lowest for particles with a size of approximately 0.2 µm. Table 1 lists the collection efficiency of air filters for suspended particles by particle size based on the ASHRAE standard. Medium‐efficiency air filters in Japan are generally used in office buildings, and air filters with a medium efficiency of 60, 75, 90, and 95% as detected by the colorimetric method (method using N‐bromosuccinimide, JIS9908: 2001) correspond to the minimum efficiency reporting value (MERV) of 11, 12, 13, and 14, respectively, as indicated by the shaded area in the table.^43^

**TABLE 1:** Minimum efficiency reporting values and filter efficiencies by particle

MERV	0.3‐1.0 µm	1.0‐3.0 µm	3.0‐10 µm	Colorimetric method
1	n/a	n/a	E_3_ < 0	‐
2	n/a	n/a	E_3_ < 0	‐
3	n/a	n/a	E_3_ < 20	‐
4	n/a	n/a	E_3_ < 20	‐
5	n/a	n/a	20 ≦ E_3_	‐
6	n/a	n/a	35 ≦ E_3_	‐
7	n/a	n/a	50 ≦ E_3_	40
8	n/a	20 ≦ E_2_	70 ≦ E_3_	40
9	n/a	35 ≦ E_2_	75 ≦ E_3_	50
10	n/a	50 ≦ E_2_	80 ≦ E_3_	50
11	20 ≦ E_1_	65 ≦ E_2_	85 ≦ E_3_	60
12	35 ≦ E_1_	80 ≦ E_2_	90 ≦ E_3_	75
13	50 ≦ E_1_	85 ≦ E_2_	90 ≦ E_3_	90
14	75 ≦ E_1_	90 ≦ E_2_	95 ≦ E_3_	95
15	85 ≦ E_1_	90 ≦ E_2_	95 ≦ E_3_	98
16	95 ≦ E_1_	95 ≦ E_2_	95 ≦ E_3_	‐

Here, the collection efficiency of MERV 13 by particle size (0.3–1.0 µm: 50%, 1.0–3.0 µm: 85%; 3.0–10 µm: 90%) as recommended by ASHRAE and the previously mentioned single‐pass collection efficiency of MERV 13 for the influenza virus, as determined from the composition ratio of the influenza virus by particle size by Noti et al., was 78%. The ventilation rate including recirculated air in a general office building is 6 ACH (outside air: 2 ACH; recirculated air: 4 ACH), resulting in a circulation of air every 10 min. Thus, it is estimated that 78% of infectious viruses with the same composition ratio by particle size were removed from the room every 10 min. Furthermore, the ventilation rate of 6 ACH, including the recirculated air, is equivalent to a clean air supply of 5.1 ACH (2 + 4 × 78%). High‐efficiency particulate air (HEPA) filters (with a particle collection rate of 99.97% or more for particles with a rated air volume of 0.3 µm) are used for rooms where air cleanliness is highly needed, such as in hospital operating rooms.

The maintenance of the air filter can be performed as usual for full outside air operation; however, for the return air operation, it is recommended that the differential pressure of the filter should be checked frequently and that the filter should be replaced earlier than usually required so as to not decrease the air volume due to the particles collected by the filter and prevent the penetration of the collected particles.

Static electricity is one of the principles of particle capture by a filter, and the electrostatic air filter strengthens this effect. When either or both the particles and the fibers of the air filter medium are charged, an electrostatic force acts between the particles and the fibers, which results in a significant increase in collection efficiency. The electret filter has a semi‐permanently polarized filter in the interior of the fiber and stable charge state, and a significantly high collection efficiency can be expected for fine particles.^44^ However, the electrostatic force is gradually lost when mist‐like particles, such as cigarette smoke, are deposited on the filter, which tends to decrease the efficiency. HEPA filters have a high‐collection efficiency but a large pressure loss; moreover, they are difficult to use for HVAC systems and portable air purifiers. Therefore, an electrostatic filter improves the collection efficiency of fine particles while reducing pressure loss. An electrostatic HEPA filter is used in portable air purifiers sold by Japanese manufacturers. Adding the effect of static electricity ensures an initial performance equivalent to that of the HEPA filter, and it is expected that its performance will satisfy the requirements of the Japan Electrical Manufacturers’ Association standard, JEM 1467.^45^

## 5 Natural Ventilation by Opening Windows

In addition to school classrooms and detached houses, there are also certain recently built high‐rise buildings in which windows or outlet openings for natural ventilation can be opened in consideration of the business continuity plan measures for the improvement of energy conservation performance. It is recommended that such openings be opened in addition to operating mechanical ventilation as long as it does not cause a draft or thermal discomfort. In particular, opening windows are recommended because securing a draft (i.e., several dozens of ACH) will achieve indoor air cleanliness equivalent to the level of outside air within a few minutes (if a volume of outside air equivalent to three times the volume of the room is taken in, then 95% of the indoor air will be replaced); therefore, it is desirable to regularly perform this action. It is important to achieve a wind pressure difference acting on the outlet opening to secure a large ventilation rate; consequently, opening outlets on different sides of the building is necessary. If there is a door between the outlets, securing an opening area equivalent to the outlet opening area is necessary so that the door will not block the ventilation.

A fan can be installed at the outlet opening to forcibly exhaust the indoor air and ventilate the room if achieving a wind pressure is difficult. In a case where a fan is installed on the indoor side at approximately 40 cm from the opening surface in a room with a floor area of approximately 60 m^2^ to exhaust the indoor air, a ventilation rate of 500 m^3^/h can be achieved under the conditions of no wind and one opening and 800 m^3^/h when there are two openings (only one fan is installed).^46^

## 6 Visualization of Indoor Air Environment

The state of ventilation in a room is difficult to sense for a person as one would with temperature or humidity; therefore, it is desirable to measure the indoor CO_2_ concentration and visualize the extent of ventilation sufficiency. This allows us to directly confirm whether a CO_2_ concentration of 1000 ppm or lower, which is the standard recommended in the Act on Maintenance of Sanitation in Buildings, is satisfied; moreover, if the standard concentration is satisfied in a steady state, then the ventilation rate would be secured at over 30 m^3^/h per person. CO_2_ monitors can be purchased online at a wide range of prices; however, some of them are entirely unreliable in terms of measurement accuracy at approximately 1000 ppm. Therefore, their performance should be verified based on the device specifications, and a device that can accurately measure approximately 1000 ppm using the nondispersive infrared method is ideal. It should also be noted that CO_2_ monitors cannot evaluate the reduction effect in infection risk for air purifiers and air filters.

## 7 Benefits of Face Masks

According to the findings up to February 2021, He et al.^47^ analyzed data from infected people and estimated an average onset interval (interval from onset of infection in a primary infected person to onset of infection in a secondary infected person) and incubation period of 5.8 and 5.2 days, respectively; moreover, they also estimated that the peak of infectious virus release was generally on or just before the onset date. Given that 44% of the primary infected individuals who infected the secondary infected individuals were presymptomatic, the results indicated the inadequate nature of postsymptomatic isolation measures and the importance of wearing masks universally, regardless of the symptoms. A research by Ueki et al.^48^ can be cited as an example that actually used SARS‐CoV‐2 to verify the protective effect of wearing a face mask. Cases where a mask was worn during inhalation and no mask was worn during exhalation demonstrated that the inhalation rate of the virus decreased by 20‐40% when a cotton mask was worn and 50% when a surgical mask was worn; however, wearing an N95 mask correctly resulted in a reduction of 80‐90%. In the next case, a mask was worn during exhalation and no mask was worn during inhalation, which demonstrated that over 50% of aerosols were blocked with cotton and surgical masks, and significant protective effects were observed with the N95 mask. The cases where masks were worn during both exhalation and inhalation demonstrated a synergistic effect on reducing inhaled viruses. Meanwhile, there were cases where a mouth or face shield is used to allow the face to be seen or because it is difficult to breathe when wearing a mask. Lindsley et al.^49^ investigated how masks, neck gaiters, and face shields affected the number of droplets released. The results showed that the capture efficiency was 99% for the N95 mask, 50‐60% for surgical masks, cotton masks, and neck gaiters, and only 2% for face shields. Therefore, the face shield does not reduce the amount of viruses that are released. Although it can be expected to block droplets produced by the other party, it is not a substitute for a mask. Therefore, it is universally recommended to wear normal masks in public areas to reduce the risk of infection.

## 8 Flushing the Toilet with the Lid Closed and the Enforcement of Ventilation

There is a possibility that aerosol particles containing the active virus are generated when an infected individual defecates in a toilet and flushes the water to dispose of the waste. In the previously mentioned research report, the flushing method and drainage characteristics of the toilet bowl were not clearly understood. However, reports using actual toilet seat surfaces showed that aerosols flew up to 25 cm away from the surface when the test toilet bowl was flushed, and computational fluid dynamics analysis showed that aerosols flew up to 106.5 cm away from the floor surface on which the toilet bowl was installed.^50^, ^51^ It is recommended that the lid of the toilet bowl be closed during flushing to reduce this effect to the maximum possible extent. Meanwhile, it has been indicated that droplets adhere to the inner surface of the lid when the toiled is repeatedly flushed with a closed lid; therefore, measures such as conducting regular cleaning and attaching a sterilizer (e.g., GUV) are necessary.^52^ Lai et al.^53^ installed an ultraviolet‐C light‐emitting diode that has a high sterilizing effect on the bacteria on the toilet seat of a toilet bowl and investigated the sterilizing effect on *E. coli*, Salmonella, *Staphylococcus epidermidis*, and other bacteria. A toilet bowl equipped with a virus sterilization device is expected to be developed in the future.

The perfect water seal of the toilet should be regularly checked, and it should be noted that an unusual odor will emerge from the drain if the trap seal is broken. Additionally, the exhaust fan of the bathroom should be operated at all times so that aerosol particles that may cause infection do not leak out and contaminate other spaces. If there is a window in the toilet area, it must not be opened because there is a risk that the air in the toilet area that contains the aerosol particles may leak outside when the window is on the upwind side.

## 9 Adjustment of Air‐conditioning Temperature and Humidity

The effects of indoor temperature and humidity, especially in terms of limiting the spread or resolving the COVID‐19 infection, can be enhanced as these parameters may affect the activity of SARS‐CoV‐2 and the transmission distance in the air. It was reported at an experimental level that SARS‐CoV‐2 activity on the surface of an object decreased when the temperature (24–35°C) and relative humidity (20%–80%) were high.^54^ Different results have been reported to date regarding the activity of the virus in air in an aerosol state. The inactivation rate of SARS‐CoV‐2 in a room at 20°C in an environment shielded from the sun was significantly higher when the relative humidity was 70%.^55^ Meanwhile, the inactivation rate at a relative humidity of 40%–60% was higher than that at a relative humidity of 68‐88% for aerosols created using artificial saliva as a result of using tissue culture media.^56^

Indoor temperature and humidity have a major effect on the transmission distance of SARS‐CoV‐2. It was reported that the distance of droplet transmission in air at a low‐temperature/high‐humidity environment was more than three times longer than that in a high‐temperature/low‐humidity environment. However, droplets in a high‐temperature/low‐humidity environment dried more quickly, forming many aerosols. These formed an aerosol particle cloud, which reached a greater distance and were suspended in air for a long period of time.^57^ Furthermore, the transmission distance of SARS‐CoV‐2 was longer in an environment with a relative humidity of 40% rather than 60%.^58^

It is also widely known that low‐temperature/low‐humidity environments adversely affect the defense mechanisms of the respiratory trachea in humans^59^, ^60^; moreover, it has been reported that it is generally desirable to control the relative humidity in the range of 40%–60% for the prevention of respiratory diseases.^14^ Therefore, temperature and humidity control measures should satisfy the management standards specified in the Act on Maintenance of Sanitation in Buildings. Specifically, the relative humidity control standard value is 40%–70%, but achieving a range of 40%–60% is ideal.

## 10 Effective Use of Air Purifier

Air purifiers for suspended particles are approximately divided into filtration and electrostatic‐collection types (air particles are charged when they pass through an ionization section, and these particles are captured by an electrostatic dust collector positioned behind the ionization section; they are primarily used in business environments). There are other types of recently developed air purifiers such as those that release ions; however, it has been reported that these technologies demonstrated inferior performance when compared with the existing filtration technology with regard to reducing the effects of active viruses suspended in air^61^; therefore, here, we describe a filtration‐type air purifier. On March 10, 2020, in their “Requests for improvement regarding the labeling of products claiming preventive effects against SARS‐CoV‐2 and alert to general consumers”,^62^ the Consumer Affairs Agency urgently requested improvements in negative ion generators and ionic air purifiers to businesses that had relevant labels on their products.

The filtration principle of the filter‐type air purifier is the same as that of the above‐mentioned air filter, but in the case of the air filter installed in the air conditioner, most of the air supply is delivered to the room through the air filter, even though there is a marginal leak. Meanwhile, the air purifier has a mechanism for filtering the suspended particles in the air while circulating the air in the room. Therefore, the purification performance of the filtration‐type air purifier is related not only to the filter capture efficiency *η*, but also to its air flow rate *q* and room volume *V*. The air purification performance of the filtration‐type air purifier is determined by the equivalent ventilation volume *qη/V*, which is equivalent to the capacity converted to the amount of outdoor air.

The Ministry of Health, Labour and Welfare and REHVA stated that HEPA filters are required when considering the filter capture efficiency of air purifiers.^13^, ^63^ The filter capture efficiency and air flow rate are important factors; therefore, the flow rate and number of air purifiers must be selected by considering the volume of the target space. The Ministry of Health, Labour and Welfare stated that "Air purifiers that use a HEPA filter" and have an air flow rate of 5 m^3^/min need to be used. The air purifier should be installed within a range of 10 m^2^ (six tatami mats) from where people are.” The capture efficiency of infectious aerosols is almost 100% if there are no leaks; therefore, the equivalent outdoor air supply becomes 12 ACH if the room volume is 25 m^3^. This value is more than double the recommended value of REHVA.^13^ As mentioned earlier, air purifiers produced in Japan often use an electret filter called electrostatic HEPA. Such a filter is effective based on the initial performance, which was confirmed to be equivalent to that of an HEPA filter. The air purifier is effective as an auxiliary equipment; however, the virus concentration reduction effect by ventilation is greater if the required ventilation rate can be achieved.

## 11 Germicidal Ultraviolet

The absorption spectrum of biological deoxyribonucleic acid (DNA) is around 254 nm (ultraviolet‐C (UVC) wavelength region: 100–280 nm); therefore, the irradiation of bacteria, fungi, and viruses with UVC rays results in DNA damage and eliminates DNA replication abilities. UVC sterilization utilizes this principle. Additionally, base sequences of single‐stranded ribonucleic acid viruses such as the influenza A virus and SARS‐CoV‐2 are destroyed and they lose their replication functions when exposed to UVC rays.^64^ The actual sterilizing effect of UVC rays is determined by the product (dose) of the ultraviolet (UV) ray intensity *I* (mW/m^2^) and the irradiation time *t* (s). The survival rate of the virus under UVC sterilization is given by the following equation. The *k* value of the coronavirus, including SARS‐CoV‐1, was reported as 1.106 cm^2^/mW·s (0.1106 m^2^/J), and its 90% sterilization dose was 2.1 mW·s/cm^2^ (21 J/m^2^).^65^ For example, if the intensity was 0.1 mW/cm^2^, then it is predicted that 90% of the virus would be inactivated in 21 s of irradiation. It can be observed that the coronavirus on a surface is easily sterilized by UVC under these time conditions. Meanwhile, when installed in an air conditioner, sterilization of viruses with UVC takes longer because the time required for the air to pass through once is short. No consensus has been reached at this time for the *k* value of SARS‐CoV‐2.

$$S_{t}=e^{‐kIt}$$

where *S_t_*: Survival rate (‐), *k*: Sterilization coefficient (cm^2^/mW·s), *I*: UV intensity (mW/cm^2^), *t*: Irradiation time (s)

GUV has been recommended owing to its effectiveness by WHO,^66^ Centers for Disease Control and Prevention (CDC),^67^ REHVA,^13^ and ASHRAE.^14^ Furthermore, the WELL Building Standard was revised in April 2020, and UV sterilization was added to the measures against microorganisms in the air relating to COVID‐19.^68^ GUV‐based methods are classified into either the up‐room or in‐duct method, depending on the installation location of the UV lamp. This refers to the installation of the UV lamp in the upper section of the room in the former case and within the air‐conditioning system (in the air conditioner or duct) in the latter case. UVC affects human health); therefore, it is important that humans should not be directly exposed to UV rays.

## 12 Notes of Caution for Resuming Equipment

The extensive spread of SARS‐CoV‐2 has resulted in the shutdown of several building equipment systems. If there is stagnant water in the air‐conditioning system (e.g., cooling tower, heat storage tank) or hot water supply system (e.g., hot water storage tank and hot water supply pipe), then the possibility of *Legionella* spp. growth and the risk of developing legionellosis have been indicated upon resumption of operations.^13^ Measures with reference to legionellosis prevention guidelines formulated by each country and organization must be implemented when resuming equipment usage.^69^

## 13 Hand Hygiene and Peripheral Hand‐Washing Equipment

Daily handwashing is one of the measures for preventing contact transmission. The “Q&A on SARS‐CoV‐2” by the Ministry of Health, Labour and Welfare stated that the number of viruses attached to hands was reduced to 1/100 when hands are washed with water for 15 s and to 1/10,000 when water is run over the hands for 15 s after washing with soap for 10 s.^70^ The effectiveness of alcohol disinfection (70‐95% ethanol) was also mentioned in situations where washing of hands was not immediately possible. A practical example of a “new lifestyle” published by the Ministry stated that carefully washing hands with soap and water for approximately 30 s is important (hand sanitizers can also be used).^71^ The COVID‐19 guidelines of the Indian Society of Heating, Refrigerating and Air Conditioning Engineers recommend the use of soap and washing of hands for at least 20 s.^72^ These guidelines only describe the recommended washing time; however, a study in Japan that examined the minimum amount of water for ensuring hygiene confirmed that bacteria disinfecting effects of 92‐98% were observed for approximately 20–30 s of washing under a water flow rate of 4.6–5.0 L/min while washing hands using a normal faucet (estimated water consumption of approximately 1.5 liters per use).^73^ Adopting a touchless faucet (or sensor faucet) that can discharge water without touching the faucet handle is desirable for preventing contact transmission in the future.

The appropriateness of using a jet dryer that dries hands after washing (i.e., hand dryer) has also been questioned, and many buildings prevent its use.^74^, ^75^ Hand dryers include a high‐speed air dryers (blow‐of effect) and warm air dryers (evaporation effect); however, according to the National Institute of Infectious Diseases New Influenza Countermeasure Plan (draft) of the National Institute of Infectious Diseases, paper towels for hand wiping are always available in toilet facilities, and the use of warm‐air‐type jet dryers is prohibited.^76^ There are also three points of concern regarding the use of high‐speed air dryers, i.e., it blows contaminated air present in the room to the hands of the individual, it scatters water droplets, bacteria, and viruses that generally adhere to hands when handwashing is insufficient at the time of use, and water droplets and bacteria adhering to the hand‐insertion section are also be scattered around the room.

Many previous studies on the diffusion of droplets and sterilization effects of hand dryers have compared the number of water and bacteria droplets that are diffused during use and the sterilization effects of high‐speed air, warm air, and paper towels. Cases in other countries have shown that the number of bacteria scattered in the surrounding air after hand drying was 4.5 times higher for the high‐speed air dryer (70.7 colony‐forming units (cfu)) than for the warm air dryer (15.7 cfu) and 7 times higher than that for paper towels (2.6 cfu); moreover, it was considered advantageous to use paper towels, which have fewer droplets.^77^, ^78^ There are similar reports in Japan related to the high‐speed air dryer; however, when comparing the research results with those of other countries, it has been indicated that the number of bacteria adhering to the hand (or gloves) at the beginning of the performance test was high in reports from other countries, and the initial conditions were different.^79^ The report stated that the test evaluation results under conditions similar to real‐life use had minimal effects on the above‐mentioned three points of concern, and detailed experimental results have also been reported.^80^, ^81^ However, it has been stated that the frequency of cleaning and disinfecting the hand‐insertion part of the hand dryer should be daily; further, the drain tank, drainage channel, and filter should be cleaned and disinfected at least once a week, but preferably more frequently.^79^ Furthermore, it is difficult to maintain the cleanliness of each part of the dryer given that people use the toilet facilities every day and its frequency of use is high; consequently, it is speculated that its use should be prohibited. Adding a GUV device and measures to suppress the diffusion of droplets should be considered in the future.

## 14 Recommended Operating Methods of Building Equipment for Each Building Application

This clause shows the desirable building equipment operation method for reducing the infection risk of the Covid‐19 in different type buildings. Although the object assumes the building and air‐conditioning / ventilation system in Japan, it is applicable also to other countries and areas in case of same climate conditions as Japan when it had the same equipment.

### 14.1 Principles of the measures to reduce risk of airborne diseases

The Wells–Riley model^82^ is often used to assess the risk of infection in respiratory diseases that are suspected to be airborne, such as SARS‐CoV‐2. The Wells–Riley model is based on the concept of the quantum of infection and uses the incidence of infectious aerosol particles to model the probability that an individual exposed to an infectious aerosol will be infected in a well‐mixed indoor environment in a steady state, as expressed by the following equation.

$$P=1‐\exp\left(‐\frac{\mathrm{Iqpt}}{Q}\right).$$

where *P*: Infection probability (‐), *I*: Number of infectors (‐), *q*: Quantum generation rate (1/h), *p*: Breathing rate (m^3^/h), *t*: Exposure time (h), *Q*:Ventilation rate (m^3^/h)

The percentage of infected individuals in Japan is not currently well understood; however, if it is assumed that it is approximately 1 in 1000 people, then in most cases, people will not be present indoors with an infected person, and the issue is related to how the risk of secondary infection can be reduced in the rare case where an infected person is present. To do so, *I* = 1 should be set in the Wells–Riley model, and the absolute value of the exponential part of the equation should be set as close to zero as possible. The principles for doing so are as follows.

Reduce *q*: Source control of infectious aerosols is important. It is said that the number of droplets generated increases proportionally with the loudness when speaking. Certain rare cases are known to exist in individuals who are super‐emitters and generate a large number of droplets, which is an order of magnitude higher than that of others^83^; moreover, it is suspected that the primary infected individual in the aforementioned chorus group cluster could be classified under this category^19^. Furthermore, according to Zhang^84^, the number of infectious aerosols generated was reduced to 5, 25, and 50% when using an N95 mask, a surgical mask, and a regular cotton mask, respectively. Sufficient knowledge has not been obtained regarding the standard quantum value of SARS‐CoV‐2, but it is said that the value is similar to that of influenza at approximately 15‐500, which is smaller than that of measles at 570‐5600^85^.

Reduce *p*: The respiratory rate is determined by the metabolic rate. Therefore, it is best to avoid exercising in a public indoor space and to stay quiet without being excited.

Reduce *t*: Reduce the time spent at a location as much as possible. Avoid long stays in risky spaces.

Increase *Q*: Here, *Q* is the sum of the amount of outside air taken in and the amount of circulating air filtered by an air filter or air purifier. The risk of infection decreases with increased ventilation.

### 14.2 Offices (central air‐conditioning system)

As a general principle, adjustments should be made to increase the intake of outside air. Attention should be paid to the air balance and the opening of the volume damper of the outside air supply fan and exhaust fan should be increased. If the air volume is controlled by an inverter, then the current utilized by the air supply and exhaust fans is increased, and the inverter is used to increase the rotation speed. The automatic control of the outside air volume should be removed, and a constant air volume system should be opened and fixed outside the air system. Alternatively, for fans driven by a motor through a pulley, the pulley diameter on the motor side is increased. The ventilation rate is increased by setting the operation mode to a particularly vigorous operation within a range that does not interfere with the generated noise. Changing the filter of the outside air system may also lead to an increase in the air volume.

For ventilation equipment in a building with CO_2_ concentration control, the ventilation rate increases when the indoor CO_2_ concentration setting value (generally around 1000 ppm) is lowered (the ventilation rate reaches a maximum when it is lower than the outside air concentration). When outside air‐cooling exists, the upper temperature limit of the outside air‐cooling condition is raised, its lower temperature limit is lowered, and adjustments are made so that the outside air‐cooling operation is prioritized. Additionally, if there is a timer‐based operation control, then adjustments should be made to extend the ventilation operation time beyond the time spent in the room, start the operation several hours earlier than usual, and delay the stop time in consideration of cases where the occupants are staying, with continuous 24‐hour operation if possible.

If there is a total heat exchanger, the static‐type heat exchanger is handled similar to the subsequently described unit air‐conditioning system. In case the exchanger is the rotary type with a purge sector, if the pressure balance is properly adjusted (i.e., pressure of return air system < pressure of supply air system), then the risk of virus leakage is considered to be low. Therefore, assuming that the operating status is confirmed and adjusted appropriately, the operation is implemented in a mode where the effective ventilation rate (airflow obtained by subtracting the leakage amount from the supply air volume) is large.

If the air volume is adjusted by a variable‐air‐volume (VAV) system, then it is speculated that the external air volume may decrease in proportion to the internal air volume. Certain buildings may install a VAV system or motor damper (air‐volume adjusting damper) in the air duct connected to the air conditioner and introduce a control that can secure the external air volume, even at the lower limit of the air supply in the VAV system on the indoor side. However, when this is not the case, it is possible to adjust the general minimum opening of the air supply of the VAV system on the indoor side of 30% to a larger opening ratio to secure the external air volume, or as an ultimate measure, to set the air volume to a constant level without controlling the air volume.

### 14.3 Offices (unit air‐conditioning systems)

Adjustments should be made such that the air flow of the outdoor air conditioner and total heat exchanger is as large as possible. It is important to confirm that the ventilation system is operating in coordination with the air‐conditioning system and air conditioning alone without ventilation must be avoided. Furthermore, the air‐conditioning system must be operated at a constant air volume instead of in the automatic operation mode to achieve a circulating air volume in the indoor unit. Similar to the central air‐conditioning system, a longer operating time of the ventilation system should be set, and it should be set to a 24‐hour operation, if possible.

In the case of a static total heat exchanger (heat exchange‐type ventilation fan), the risk of virus leakage through the heat exchange element is low, and only 5% of the virus leakage stems from the outside air and return air; consequently, there is no problem in operating in the heat exchange mode; however, the instruction manual should be checked for the appropriate operation mode in which the effective ventilation rate increases by increasing the processing airflow.

The performance of the air filter is often inferior to that of the central‐type air‐conditioning system; moreover, it is difficult to substantially increase the airflow through filtration. Therefore, in case of a concern, an additional filtration‐type portable air purifier can be installed or the indoor air filter performance can be upgraded by using a medium‐efficiency filter (generally available as an optional specification).

Additionally, when the drain generated from the indoor unit during the air‐conditioning operation is treated along with the common drainage system, ventilation may exist between the rooms and the drainage system after merging through the drainage pipe, resulting in the diffusion of contaminated air. Installing a check valve for airflow in the drainpipe of an indoor unit is effective in preventing this.

### 14.4 Residences

#### 14.4.1 Infection risk and measures for ventilation equipment

It is important to avoid contracting an infection outside as well as bringing the virus into the home to prevent domestic infections. According to He et al.,^47^ approximately half of the primary infected individuals infected the secondary infected individuals before the onset of symptoms; therefore, it can be noted that COVID‐19 is infectious even before the onset of illness, as mentioned earlier. It is speculated that infected individuals spread the disease to cohabitants through close contact from one person to another without being detected during the asymptomatic period; therefore, it is difficult to prevent infection even if measures are taken once the symptoms appear.

If the risk of infection at home is low, then special measures need not be taken at home if measures to counteract infection have already been taken outside and hand hygiene is performed when returning home. However, infection countermeasures at home should be strengthened if there is a risk of infection, such as when there are family members or visitors who may have been infected outside the home. Additionally, special measures are required if infected individuals are cohabiting or if they have been in close contact with sickbeds in a medical institution owing to urgent circumstances.

#### 14.4.2 Measures when using ventilation equipment in case of a risk of infection

**FIGURE 2:**
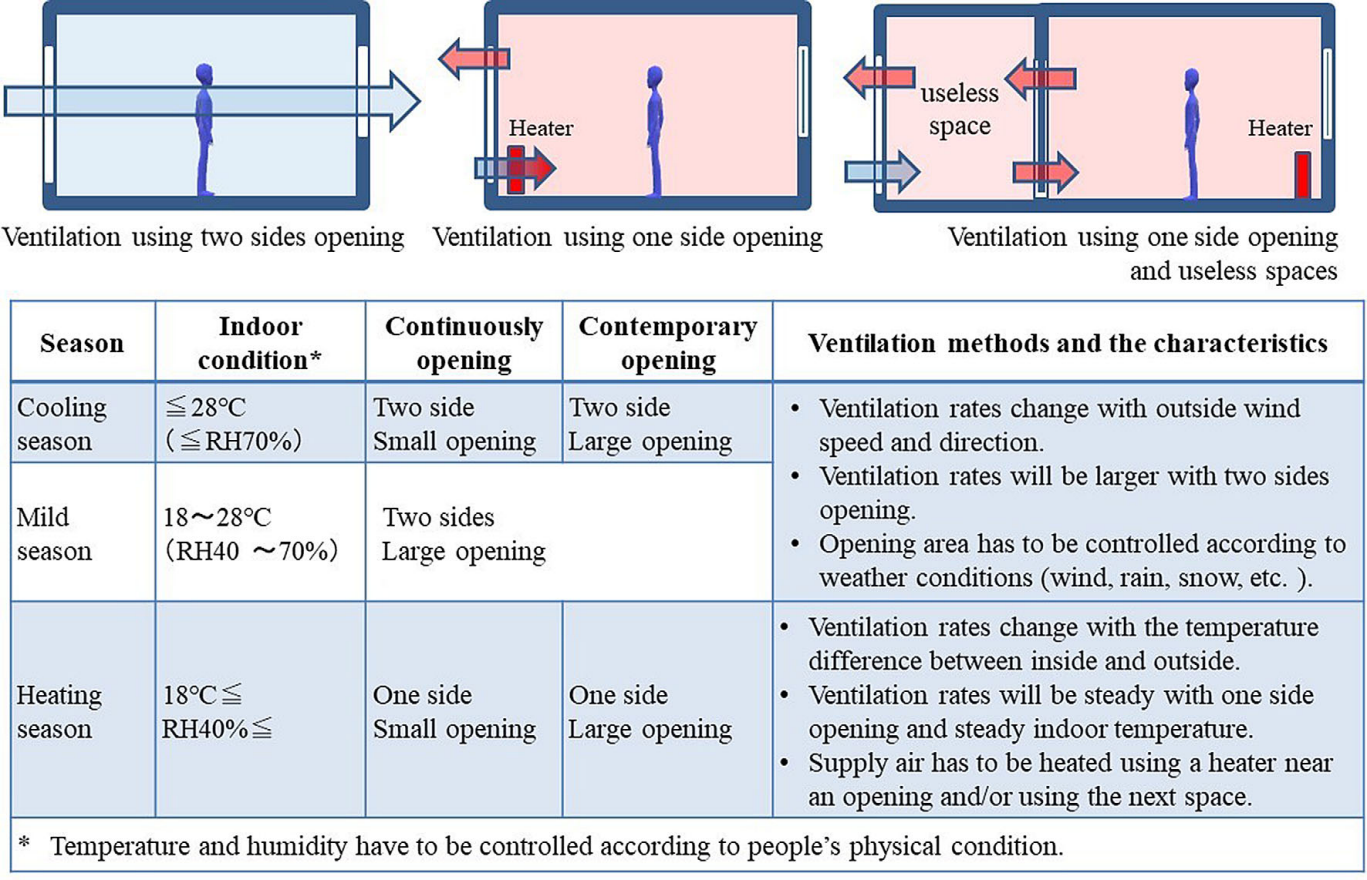
Window opening methods for infection control of measures for ventilation equipment when infected people and people in close contact are in the waiting period

Even in cases where the infection risk is low, a constant 24‐hour ventilation system should be maintained. The ventilation fan of the bathroom or toilet should always be operated in the absence of a 24‐hour ventilation system. Filter maintenance must be performed. When there is a risk of infection, the ventilation volume should be increased by using window ventilation, an air purifier, or a kitchen ventilation fan in addition to the constant operation of the ventilation equipment. For ventilation through open windows, a method to open windows that uses heating and cooling equipment should be devised so that the indoor environment does not deteriorate and the different weather conditions (wind, rain, snow, etc.) and external environment (noise, air pollution, etc.) are considered, as shown in Figure 2.^59^, ^63^

As a general rule, infected people should be isolated in hotels and hospitals. However, there are times when people have to take care of themselves at home, such as when waiting for polymerase chain reaction test results or until hospitalization arrangements are completed. The AIJ has introduced a method of using plastic curtains to separate the living space between infected persons and their families, as well as establishing negative pressure in the zone of the infected individuals by using the exhaust fan of the toilet; moreover, all precautions should be taken to further reduce the risk of aerosol infection.^86^

### 14.5 Schools

Classrooms in schools are important because they are rooms where people stay for long periods of time. Sufficient ventilation measures must be taken after implementing social distancing. Consequently, classrooms should be utilized to only half its capacity; however, if this is difficult to incorporate, then measures such as masks, face shields, and room partitions are required. If the building is not specified by the Act on Maintenance of Sanitation in Buildings, the ventilation rate of the mechanical ventilation equipment is often insufficient, and window ventilation is required. The AIJ provides information on the relationship between the open area of the windows/doors for single‐corridor or mid‐corridor classrooms and classroom capacity based on the actual measurements of the total open area of the windows/doors of the classroom and the ventilation rate^87^; therefore, this should be used as a reference. However, objective evaluations of the degree of ventilation sufficiency are difficult to perform when only relying on natural ventilation; therefore, the quality of the indoor air environment should be visualized using a CO_2_ monitor. If possible, the ventilation rate should also be adjusted to below 1000 ppm based on the Act on Maintenance of Sanitation in Buildings, or at least below 1500 ppm under the current school environmental hygiene standards.

### 14.6 Cinemas/theaters

In closed spaces such as cinemas and theaters, the occupied area per person is crowded at 0.5–1 m^2^; however, it is speculated that the number of droplets would be small if the film is watched quietly. Acquiring a larger occupied area would be effective in ensuring a sufficient ventilation rate and social distancing per person. Furthermore, the risk can be reduced by providing a large amount of ventilation at the time of rotating people in and out of the area.

### 14.7 Izakaya/Karaoke bars

The act of yelling and singing has the risk of generating a large number of droplets; consequently, taking protective measures against droplets is important. Efforts to reduce droplet concentration are made by providing a large amount of ventilation, and ensuring social distancing is a basic measure. Microphones and music selection controllers are likely to be sources of contact transmission and require frequent disinfection.

## 15 Conclusion

In this paper, in order to reduce the risk of infection with COVID‐19 in the buildings, we summarized the desirable operation methods of air conditioning and sanitary equipments based on the knowledge about the characteristics of SARS‐CoV‐2 and the outbreak cases that have been obtained so far. The target is assumed to be a Japanese building, it is applicable also to other countries and areas in case of similar climate conditions as Japan when it had the same equipment. From the findings obtained so far, it is almost certain that the risk of outbreaks is high in a closed space with poor ventilation, so at least 30 m^3^/h of outside air per person recommended by the Ministry of Health, Labor and Welfare should be ensured, and further measures should be taken to reduce the risk of infection. Specifically, it is recommended to consider the following points.

1. The air conditioning system should be operated and adjusted to increase the amount of outside air as much as possible.
2. Use an air filter or air purifier with as high performance as possible.
3. Open windows for natural ventilation as long as possible unless there is problem in terms of draft and thermal environment.
4. Visualize the ventilation conditions with a CO_2_ monitor. However, CO_2_ monitors cannot evaluate the risk reduction effect of air filters and air purifiers.
5. Thorough universal masking in the room and use surgical masks if possible.
6. Toilet should be flushed with the lid closed, the exhaust fan should be operated at all times, and the water seal should be checked regularly.
7. The room temperature and humidity should be kept within a comfortable range for the occupants, and the relative humidity in the room should be controlled to 40%–60%.
8. Note that there is a possibility of Legionella spp. Propagation due to water stagnation when the building operation is resumed.
9. Keep in mind the hygiene of hands and fingers by washing them and pay attention to ensuring the hygiene of handwashing machines and peripheral devices.

## Disclosure

The authors have no conflict of interest.

## Funding information

No funding information is provided.

## Data Availability

Data sharing in not applicable to this article as no new data were created or analyzed in this study.
